# Outcome of phacoemulsification in patients with open-angle glaucoma after selective laser trabeculoplasty

**DOI:** 10.1371/journal.pone.0238394

**Published:** 2020-09-24

**Authors:** Yuli Park, Kyong Jin Cho

**Affiliations:** 1 Department of Ophthalmology, Dankook University Hospital, College of Medicine, Dankook University, Cheonan, Republic of Korea; 2 Beckman Laser Institute Korea, Dankook University, Cheonan, Republic of Korea; LV Prasad Eye Institute, INDIA

## Abstract

**Purpose:**

To investigate the outcome of phacoemulsification in selective laser trabeculoplasty (SLT)-treated eyes.

**Methods:**

This retrospective study included patients who had open angle glaucoma (OAG) with previous SLT who underwent phacoemulsification. We evaluated intraocular pressure (IOP), length of glaucoma control without treatment, and antiglaucoma medication or surgery. SLT-treated eyes that did not receive phacoemulsification were retrospectively chosen as a control. We investigated factors related to outcome of phacoemulsification by multivariate analysis.

**Results:**

42 eyes with previous SLT that underwent phacoemulsification and 40 controls were retrospectively evaluated. Phacoemulsification was performed 52 ± 15 months after SLT. After a mean follow-up of 74 ± 21 months, mean IOP was significantly decreased in the phaco group by 2.2 ± 2.7 mmHg (*p* < 0.001). In the SLT group, mean IOP was decreased by 0.8 ± 2.8 mmHg (*p* < 0.001). 9 eyes (16.7%) in the phaco group and 11 eyes (19.0%) of the SLT group needed topical treatment, and no eye needed glaucoma surgery in both groups. The factor related to success was higher baseline IOP (*p* = 0.002).

**Conclusion:**

Prior SLT didn’t negatively influence phacoemulsification in patients with OAG. Phacoemulsification lowered IOP effectively and safely in OAG patients who were treated with SLT.

## Introduction

Glaucoma is a progressive neurodegenerative optic neuropathy marked by the morphological changes of optic disc and results in an irreversible visual field defect by loss of retinal ganglion cells, leading to blindness [1, 2]. The treatment aims to lower the intraocular pressure (IOP) to slow the damage of optic nerve [3]. The treatment involves topical antiglaucoma medications, laser, and glaucoma surgery.

Selective laser trabeculoplasty (SLT) utilizes 532 nm, frequency-doubled, Q-switched, neodymium-doped yttrium aluminum garnet (Nd:YAG) laser that particularly aims pigmented trabecular meshwork (TM) cells without damaging adjacent structures [4–6]. The turnover of extracellular matrix and stimulation of cellular production, dislodging of trabecular cells, mechanical distension of Schlemm’s canal have been thought to be the mechanisms of SLT [7]. SLT causes less damage than unlike argon laser trabeculoplasty (ALT) and no coagulative effects on TM [8, 9]. SLT has been established for over a decade as a successful treatment for IOP reduction in open angle glaucoma (OAG) patients [10]. It is safe and potentially repeatable and can be considered in those patients who cannot tolerate drops or whose IOP is not controlled with drops sufficiently well.

Phacoemulsification is well-established as the main surgical procedure for cataract worldwide. It is associated with IOP changes postoperatively in both OAG [11] and angle closure glaucoma (ACG) [12], although ACG patients benefit significantly more than OAG patients with respect to sustained postphaco IOP reduction. Cataract frequently coexists in patients with OAG. It is important to ascertain, whether the IOP would be affected adversely in patients previously treated with SLT, if they have phacoemulsification. This is particularly pertinent, when informing patients who are obviously curious if their IOP control would suffer following phacoemulsification.

The purpose of our study is to assess the IOP control following cataract surgery in OAG with well-controlled IOP previously achieved with SLT alone. This would provide valuable information to the surgeon as to the likelihood of further IOP control that might be required after phacoemulsification.

## Materials and methods

### Study design and participants

This retrospective, comparative study evaluated patients with OAG from 2009 to 2019. Our study adhered to the tenets of the Declaration of Helsinki for research involving human subjects and followed all guidelines for investigation in human subjects required and approved by the Institutional Review Board of Dankook university hospital (IRB#DKUH202003006).

Patients were identified by scheduling records for glaucoma laser clinic. All included eyes required best corrected visual acuity (BCVA) of >20/40 and we included only subjects with open angles on gonioscopy. Other inclusion criteria were previous SLT performed for OAG, glaucoma control without topical therapy (IOP <18mmHg), and refraction between +3D and − 5D. Patients were excluded if they had history of ocular trauma, media opacity, retinal diseases, optic nerve disease other than glaucoma, history of a cerebrovascular event or systemic medication use that could affect the visual field (VF) and IOP, or systemic disease such as diabetes mellitus or hypertension. Eyes with consistently unreliable VFs which had fixation losses more than 20%, and the false-positive and false-negative errors more than 15% were excluded. Exclusion criteria also consisted of patients who were unable to have SLT successfully performed, or lost to follow up, ≤18 years old, and had a history of prior ocular surgeries.

A group of consecutive SLT-treated eyes that did not receive phacoemulsification was retrospectively hosen and used as a control. Controls were matched by the period when SLT was performed to have a similar follow-up. The same inclusion criteria and exclusion criteria applied for the study group were applied for the control group. Glaucoma therapy was prescribed in the presence of IOP >18mmHg or worsening of VF.

Ocular examinations were performed before and after the cataract surgery, at 1 month, at every 3 months in the first year, and at every 6 months in the following years. Glaucoma control was evaluated by comprehensive ophthalmologic evaluation including BCVA, slit-lamp biomicroscopy, Goldmann applanation tonometry, gonioscopy, stereoscopic examination of the optic nerve head, standard automated perimetry (SAP) using the 24–2 Swedish Interactive Threshold Algorithm (SITA) standard program (Humphrey Visual Field Analyzer; Carl Zeiss-Meditec Inc., Dublin, CA, USA), retinal nerve fiber layer thickness was determined by Cirrus OCT (Carl zeiss, Jena, Germany), and color disc photography. IOP reading was taken in a masked fashion and it was taken 3 times to get an average.

### SLT

SLT was performed with the Lumenis Selecta II (Santa Clara, CA, USA) which is a Q-switched Nd:YAG laser producing a single 532 nm wavelength pulse with a 400μm spot size and 3ns pulse duration. Initial power settings were between 0.9–1.1 mJ and were titrated until champagne bubbles were creatved. An Ocular Hwang-Latina 5.0 single mirror SLT lens (Bellavue, WA, USA) was used to visualize angle structures. All eyes received a single session of 360° laser treatment not overlapping laser spots along the TM. Post-procedure IOP was checked at 1 hour after SLT to detect early postprocedure IOP spikes.

### Cataract surgery

All patients were operated on by one experienced surgeon and cataract surgery was performed under topical anesthesia. Clear corneal incision was made and the phacoemulsification time was 1 to 2 minutes. An intraocular lens was inserted in the posterior chamber. In all cases, no intraoperative complication happened. In the postoperative period, the patients instilled antibiotics and steroid eye drops four times daily for 1 month postoperatively and tapered.

### Statistical analysis

Baseline and postoperative IOP in operated eyes were compared by the paired t-test. To assess factors related to IOP reduction, we used multivariate analysis. All of statistical analyses were performed using SPSS version 20.0 (SPSS, Inc., Chicago, IL, USA) and *p* value of <0.05 was considered to be statistically significant.

## Results

Our study evaluated 82 eyes of OAG. 42 eyes of 42 patients treated by SLT and then had received an uncomplicated phacoemulsification (phaco group) and 40 eyes of 40 patients treated only by SLT and didn’t go through cataract surgery (control group) were included in the study. There was not a statistically significant difference between groups in gender, age, BCVA, and central corneal thickness (CCT) (*p* > 0.05). The demographic and clinical characteristics of subjects are summarized in Table 1.

**Table 1:** Baseline demographic, clinical, and biometric characteristics of the subjects.

Clinical parameters	SLT+Phaco (n = 42)	Only SLT (n = 40)	*p* value
Age at cataract surgery (years)	65.3 ± 10.1	60.8 ± 9.7	0.42
Sex (M:F)	20:22	19:21	0.57
Anterior chamber depth (mm)	3.3 ± 0.4	3.1 ± 0.3	0.36
Central corneal thickness (μm)	535.1 ± 36.8	540.6 ± 33.5	0.32
Axial length (mm)	23.6 ± 1.9	23.1 ± 1.7	0.84
Spherical equivalent (D)	-0.9 ± 2.4	-0.7 ± 2.1	0.56
Average keratometry (D)	45.1 ± 2.5	44.9 ± 2.2	0.49
Mean deviation (dB)	-5.3 ± 4.7	-4.8 ± 4.1	0.37
Pattern standard deviation (dB)	3.9 ± 3.5	3.1 ± 2.8	0.54
Time from SLT to cataract surgery (months)	52 ± 15	50 ± 14	0.26

Values are presented as mean ± SD

In the phaco group, mean age at phacoemulsification was 65.3 ± 10.1 years. Mean time from SLT to phacoemulsification was 52 ± 15 months and mean follow-up after phacoemulsification was 74 ± 21 months. In the control group, mean age at the study entry (corresponding to time of phacoemulsification in the phaco group) 60.8 ± 9.7 years. Mean time from SLT to the study entry was 50 ± 14 months and mean follow-up after phacoemulsification was 76 ± 23 months. In the phaco group, mean deviation and pattern standard deviation at phacoemulsification was -5.3 ± 4.7, 3.9 ± 3.5 dB, respectively. In the control group, mean deviation and pattern standard deviation at the study entry (corresponding to time of phacoemulsification in the phaco group) was -4.8 ± 4.1, 3.1 ± 2.8 dB, respectively.

**Fig 1:**
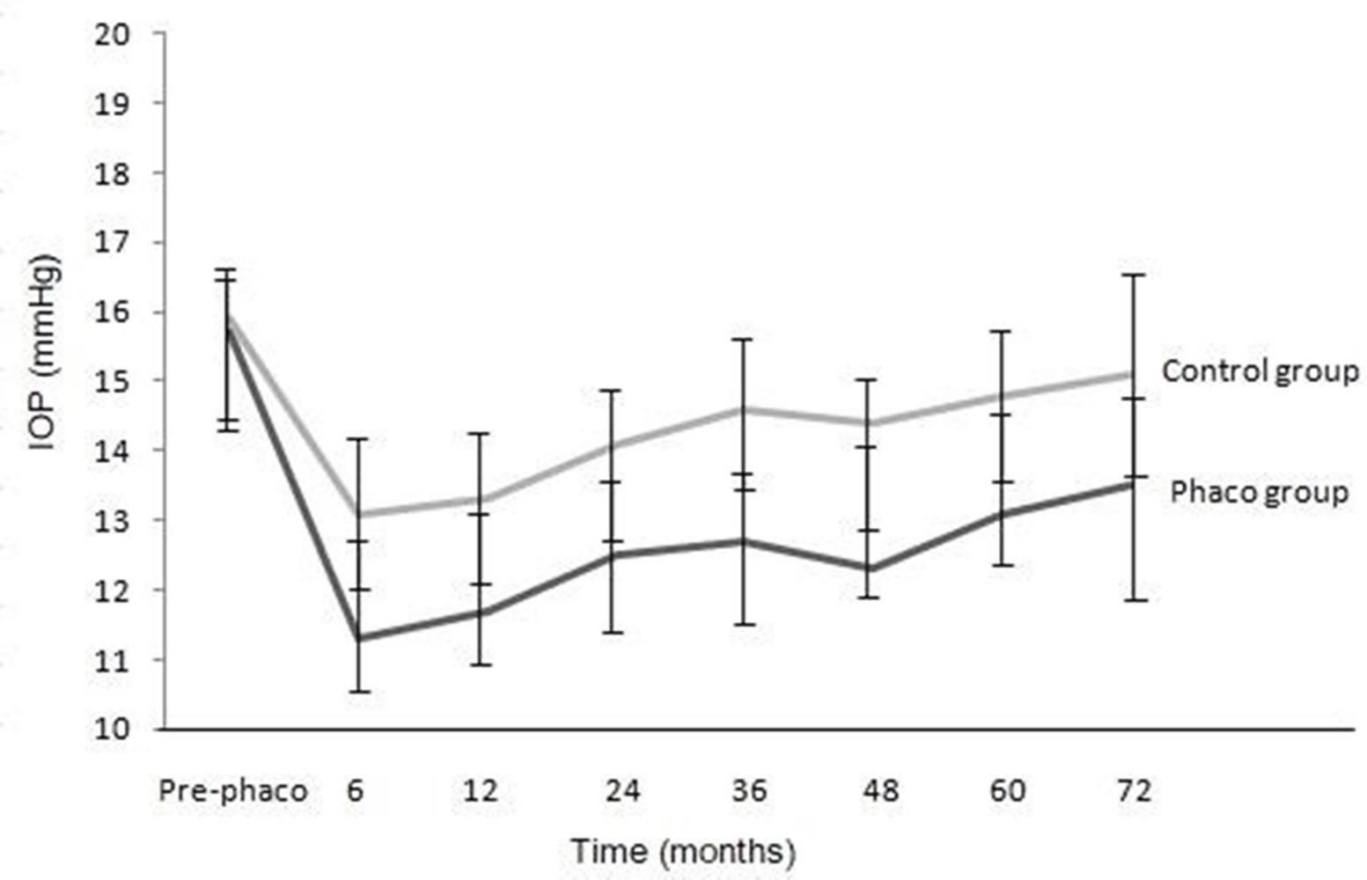
Mean intraocular pressure (IOP) over 6 years including baseline pre-phaco IOP for the phaco and the control groups.

Mean preoperative IOP was 15.7 ± 2.5 mmHg and 15.9 ± 2.6 mmHg for phaco and control groups, respectively (*p* = 0.36). In the phaco group, mean IOP at postoperative 6 years measured 13.5 ± 2.1 mmHg and in eyes without previous phaco, mean IOP at postoperative 6 years measured 15.1 ± 2.4 mmHg. In the phaco group, mean IOP had significantly decreased by 2.2 ± 2.7 mmHg (*p* < 0.001). In the SLT group, mean IOP was decreased by 0.8 ± 2.8 mmHg (*p* < 0.001). In two groups, 9 eyes (16.7%) in the phaco group and 11 eyes (19.0%) of the SLT group had required topical antiglaucoma medication to control glaucoma, although no eye had required glaucoma surgery in both groups. In the phaco group, 17% of OAG eyes showed postoperative IOP increase as with 13% eyes with an IOP decrease, whereas most of the eyes had a stable IOP as defined as a IOP variation < 5 mmHg. The overall mean IOP at preoperative, postoperative 6 months, 1, 2, 3, 4, 5, and 6 years is shown in Fig 1.

Multivariate analysis revealed that factor related to the success (no need of glaucoma therapy) was higher preoperative IOP (*p* = 0.03). No statistically significant differences in post-phaco IOP or percent IOP reduction were observed based on gender within the phaco group.

## Discussion

SLT has become popular trabeculoplasty laser to lower IOP in patients with OAG. The most significant benefit of SLT is that it produces less damage to the targeting structure allowing effective repeated lasers, which is unfeasible with ALT [10, 13]. SLT utilizes the nanosecond pulse duration that avoids collateral thermal damage since it is less than the thermal relaxation time of the melanin chromophore [13]. Another benefit of SLT is a decrease in diurnal IOP fluctuation which has been revealed as an independent risk factor for progression of glaucoma [14, 15].

In our study at each time point following phacoemulsification, IOP was reduced compared to baseline for the 6-year period of follow-up. The interval period between SLT and phacoemulsification did not affect subsequent IOP control. It was very encouraging that even those cases where SLT had been performed 2 to 3 years earlier, the IOP control was sustained after phacoemulsification. A significant remark is that none of these patients needed glaucoma surgery and successful IOP lowering was obtained following phacoemulsification. It is known that inflammatory mediators, including cytokines, are upregulated following SLT [16]. There is an evidence that it also occurs following cataract surgery and intraocular surgery generally, and this might explain the additional sustained IOP reduction that was observed after phacoemulsification [17, 18]. Several mechanisms are concerned in postoperative IOP reductions which are biochemical changes with the TM cellular response to ultrasound [19], the washout of the TM during phacoemulsification [20], a widening of the anterior chamber angle advancing TM access [21] or changes in the uveal tract enhancing outflow [22].

Chen et al. demonstrated 13% IOP reduction in eyes with primary open angle glaucoma (POAG) following phacoemulsification alone [23]. But other reports revealed considerable variability of outcomes with postoperative IOP reduction ranging from 7 to 22% [24, 25]. The different study designs and the variability of inclusion and exclusion criteria may account for this.

In our study, mean IOP had significantly decreased by 2.2 ± 2.7 mmHg in the phaco group, and in the SLT group mean IOP was decreased by 0.8 ± 2.8 mmHg at postoperative 6 year follow-up. Individual variations revealed postoperative IOP was stable in the majority of patients. Previous study showed IOP reduction of 2.3 mmHg [23]. Mierzejewski et al. demonstrated the highest reduction of postoperative IOP which is 4 mmHg after phacoemulsification in eyes with POAG, however pseudoexfoliative glaucoma was included, and because no specific gonioscopic grading was shown, eyes with narrow angle before phacoemulsification can clarify the substantial reduction in postoperative IOP after surgery [25]. Other studies included eyes with normal tension glaucoma (NTG) or medically uncontrolled POAG [26, 27]. These methodological variations possibly account for the high variability in the outcomes.

Previous studies showed 6–26% of patients had increase in postoperative IOP and 4–26% of patients needed increase in IOP-lowering medication at 1–5 years following phacoemulsification [23–28]. In this present study we found 17% OAG eyes with a postoperative IOP increase as with 13% eyes with an IOP decrease, whereas most of patients showed a stable IOP. We tried to spot eyes which showed the greatest IOP increases or decreases after surgery. The preoperative IOP was the only factor related to the variation of the postoperative IOP. As Slabaugh et al. reported [29], our study also discovered the reduction of postoperative IOP was negatively correlated with preoperative IOP in OAG patients. The timing of the phacoemulsification did not make a difference in postoperative IOP decrease. In contrast to narrow angle glaucoma, the access to the TM is excellent, and unlike in pseudoexfoliative glaucoma no abnormal stuff that needs to be washed out during phacoemulsification exists. Thus, the postoperative IOP decrease may be accounted for an enhanced function of the TM rather than improved access. Wang et al. suggested that ultrasound during phacoemulsification created an IOP lowering biochemical response on TM cells [19]. Berdal et al. proposed the posterior lens shift following phacoemulsification relaxed ciliary muscle altering the TM structure and enhancing outflow [30]. They also suggested that the growth of crystalline lens may shift the uveal tract anteriorly, compressing Schlemm canal thus reducing aqueous humor outflow. The eyes with higher preoperative IOP could have larger shift of the uveal tract because of the growth of crystalline lens resulting in greater compression of the Schlemm canal and therefore these patients could gain a greater advantage after cataract surgery. Strenk et al. revealed that pseudophakic eyes showed a posterior uveal shift by analyzing magnetic resonance images [22].

Some limitations exist in this study. First, the study design is retrospective. Second, it contains a limited number of eyes. A prospective study with a larger number of patients would be advantageous. Finally, our study involved steroids as a postoperative eye drop but it is unlikely to have had an effect on postoperative IOP, given the short duration of use.

Our study revealed that for SLT-treated OAG patients, phacoemulsification resulted in a clinically significant IOP reduction after 6 years of follow-up. According to our findings, prior SLT didn’t negatively influence phacoemulsification in patients with OAG.

In conclusion, phacoemulsification lowered IOP effectively and safely in OAG patients who were treated with SLT.
